# Resting state EEG as biomarker of cognitive training and physical activity’s joint effect in Parkinson’s patients with mild cognitive impairment

**DOI:** 10.1186/s42466-023-00273-5

**Published:** 2023-09-14

**Authors:** Carlos Trenado, Paula Trauberg, Saskia Elben, Karina Dimenshteyn, Ann-Kristin Folkerts, Karsten Witt, Daniel Weiss, Inga Liepelt-Scarfone, Elke Kalbe, Lars Wojtecki

**Affiliations:** 1 Center for Movement Disorders and Neuromodulation, Departmemt of Neurology, University Clinic Duesseldorf, Duesseldorf, Germany; 2 Institute of Clinical Neuroscience and Medical Psychology, Medical Faculty, Heinrich Heine University, Duesseldorf, Germany; 3 Departmemt of Neurology and Neurorehabilitation, Hospital Zum Heiligen Geist, Academic Teaching Hospital of the Heinrich-Heine-University Duesseldorf, Von-Broichhausen-Allee 1, 47906 Kempen, Germany; 4 Max Planck Institute for Empirical Aesthetics, Frankfurt am Main, Germany; 5 Department of Medical Psychology | Neuropsychology & Gender Studies, Center for Neuropsychological Diagnostic and Intervention (CeNDI), Faculty of Medicine and University Hospital Cologne, University of Cologne, Kerpener Str. 62, 50937 Cologne, Germany; 6 Department of Neurology, University Hospital Schleswig-Holstein, Christian-Albrechts-University, Arnold-Heller- Str. 3, 24105 Kiel, Germany; 7 Research Center Neurosensory Science, Carl von Ossietzky University Oldenburg, Heiligengeisthöfe 4, 26121 Oldenburg, Germany; 8 German Center of Neurodegenerative Diseases (DZNE) and Hertie Institute for Clinical Brain Research, Department of Neurodegenerative Diseases, University of Tübingen, Hoppe-Seyler-Str. 3, 72076 Tübingen, Germany; 9 IB Hochschule für Gesundheit und Soziales, Paulinenstr. 45, 70178 Stuttgart, Germany

**Keywords:** Cognitive decline, Mild cognitive impairment, Neurovitalis, Parkinson’s disease, Diagnostic marker, electroencephalogram, non-pharmacological, intervention, physical activity

## Abstract

**Background:**

Cognitive decline is a major factor for the deterioration of the quality of life in patients suffering from Parkinson’s disease (PD). Recently, it was reported that cognitive training (CT) in PD patients with mild cognitive impairment (PD-MCI) led to an increase of physical activity (PA) accompanied by improved executive function (EF). Moreover, PA has been shown to alter positively brain function and cognitive abilities in PD. Both observations suggest an interaction between CT and PA.

**Objectives:**

A previous multicenter (MC) study was slightly significant when considering independent effects of interventions (CT and PA) on EF. Here, we use MC constituent single center data that showed no effect of interventions on EF. Thus, this exploratory study considers pooling data from both interventions to gain insight into a recently reported interaction between CT and PA and provide a proof of principle for the usefulness of resting state EEG as a neurophysiological biomarker of joint intervention’s effect on EF and attention in PD-MCI.

**Methods:**

Pre- and post-intervention resting state EEG and neuropsychological scores (EF and attention) were obtained from 19 PD-MCI patients (10 (CT) and 9 (PA)). We focused our EEG analysis on frontal cortical areas due to their relevance on cognitive function.

**Results:**

We found a significant joint effect of interventions on EF and a trend on attention, as well as trends for the negative correlation between attention and theta power (pre), the positive correlation between EF and alpha power (post) and a significant negative relationship between attention and theta power over time (post-pre).

**Conclusions:**

Our results support the role of theta and alpha power at frontal areas as a biomarker for the therapeutic joint effect of interventions.

## Introduction

Forgetfulness, inability to sustain attention and organize ideas are among the cognitive impairments that crucially affect the life quality of patients suffering from Parkinson’s disease (PD), which represents the second most common neurodegenerative disorder, affecting > 1% of the population ≥ 65 years of age and with a prevalence set to double by 2030 [1]. It has been estimated that approximately 40% of PD patients suffer from mild cognitive impairment [2]. Although, it is commonly assumed that prevalence and severity of cognitive impairment increase as the disease progresses, 32% of PD patients already show signs of mild to moderate cognitive impairment by the time of diagnosis [2], while signs of cognitive impairment are even known in the case of prodromal PD patients.

Focusing on the mechanism of PD, it has been stated that basal ganglia-thalamo-cortical-circuits play an important role not only in motor, but also in cognitive and behavioral dysfunctions. In particular, frontostriatal and orbitofrontal loops seem to be involved in cognitive processes and behavioral flexibility. Among the most common cognitive deficits in Parkinson’s patients with mild cognitive impairment (PD-MCI) are attention and memory deficit as well as deficiencies in visuospatial perception, language and executive function [3]. Since effective pharmacological interventions to deal with PD-MCI are limited, non-pharmacological interventions such as cognitive training (CT), physical activity (PA), non-invasive brain stimulation, social engagement, and neuro-feedback represent alternative options.

With regard to CT, we previously demonstrated improvement of executive function (EF) in PD-MCI induced by multi-domain group CT, specially showing an enhanced effect for patients that were more affected [4]. Likewise, such intervention was able to improve memory function after 6 months, although the improvement was not present after 12 months, while EF remained stable in the long term [5]. Notoriously, it has recently been reported that CT increases PA in patients with PD-MCI, possibly due to effects on EF [6]. Crucially, PA has also been shown to alter positively brain function and cognitive performance [7] as well as motor symptoms [8] in PD.

Based on a multicenter (MC) study addressing the independent effect of CT and PA on cognition, we previously reported a statistical trend on the interaction between time and group (CT and PA) on overall EF and a significant effect only on phonemic fluency as a specific part of EF [4]. Moreover, we reported a significant effect on the interaction between time and group (CT and PA) on memory after 6 months of intervention, but not on EF after 6 and 12 months of intervention although EF enhancement occurred immediately after intervention [5]. Note that the MC study is slightly significant regarding the independent effect of interventions on EF, however the single center data that we consider in the present study showed no independent effect of interventions on EF, possibly due to underpower. Consequently, this exploratory study considers pooling data from both interventions to increase statistical power and provide a proof of principle for the use of resting state EEG as a neurophysiological biomarker of joint intervention’s effect on EF and attention in PD-MCI. By pooling data, we also aim to gaining neurophysiological insight into a recently reported interaction between CT and PA, that is, CT leads to increased PA possibly through EF [6] and the fact that PA favors cognitive and motor functions [7, 8]. Because MCI in PD has been shown to affect low (delta and theta) [9] and high (alpha and beta) frequency bands [10] of recorded MEG and EEG activity, we expected a relationship between cognitive ability and EEG power at those frequency bands as modulated by the applied interventions.

## Methods

### Patients

Nineteen PD patients (age range: 50–80 years) were recruited from the movement disorders unit of the University Hospital Düsseldorf. Patient inclusion criteria were: (1) PD diagnosis according to the UK Brain Criteria, (2) self-reported cognitive impairment assessed with the subjective cognitive impairment (SCI) questionnaire and/or objective cognitive impairment assessed with the Montreal Cognitive Assessment (MoCA) < 26 points, (3) PD-MCI according to Movement Disorders Society (MDS) Task Force Level-II criteria (cognitive impairment in at least two cognitive tests; *z*-score ≤ − 1, SD below the mean normative score), (4) PD duration ≥ three years, (5) stable medication within four weeks before screening and (6) written informed consent. Table 1 describes demographic, clinical and neuropsychological characteristics of PD-MCI patients that participated in the present study.

**Table 1:** Baseline demographic, clinical and neuropsychological characteristics of PD-MCI patients considered in this study (n = 19). Values are presented as the mean ± standard deviation or median and range or frequency with percentages. For baseline comparison between groups, *p*-values of Mann-Whitney-U tests, independent sample t-tests or χ^2^-tests are reported as appropriate. Self-reported activity level: 0 = “not at all active”; 1 = “little active”; 2 = “moderate active”; 3 = “very active”

	Cognitive training (*n* = 10)	Physical activity (*n* = 9)	*p* value
Age (years)	62.5 ± 1.61	61.44 ± 2.4	0.356
Sex			0.089
*Male (%)*	9 (90%)	5 (55.6%)	
*Female (%)*	1 (10%)	4 (44.4%)	
Years of education	13.8 ± 0.8 (11.00–18.00)	14.22 ± 1.12 (10.00–20.00)	0.760
Age at PD symptom onset (years)	51.9 ± 3.04	52.11 ± 2.51	0.939
Age at PD diagnosis (years)	54.6 ± 2.0	53.11 ± 2.51	0.646
PD duration (months)	96.5 ± 11.93 (48.00-188.00)	97.33 ± 15.35 (44.00-163.00)	0.905
Hoehn-Yahr stage			0.303
*1 (%)*	2 (20%)	5 (55.6%)	
*2 (%)*	6 (60%)	4 (44.4%)	
*2.5 (%)*	1 (10%)	0	
*3 (%)*	1 (10%)	0	
*4 (%)*	0	0	
UPDRS-III	16 ± 0.2.17	13.11 ± 2.11	0.966
MoCA (max. 30 points)	25.3 ± 0.33	26.22 ± 0.55	0.160
SCI – number of impaired cognitive domains (max. 6 points)	3.4 ± 0.54	2.00 ± 0.44	0.065
BDI-II (max. 63 points)	9.4 ± 1.56	6.22 ± 1.23	0.134
GSE (max. 40 points)	27.6 ± 1.71	34.00 ± 0.94	0.006
PD-MCI subtype			
*Single-domain PD-MCI (%)* *Multi-domain PD-MCI (%)*	0	0	
	10 (100%)	9 (100%)	
Physiotherapy at baseline	8 (80%)	7 (77.8%)	0.906
Cognitive training previously	1 (10%)	1 (11.1%)	0.937
Self-reported activity level^c^			
*not active at all*	0	0	
*little active*	0	1 (11.1%)	
*moderate active*	7 (70%	6 (66.7%)	
*very active*	3 (30%)	2 (22.2%)	

This study was conducted in accordance with the ethics committee of the medical faculty of the Heinrich Heine University Düsseldorf (Reg. 2,016,034,986). All patients signed a written consent for participation. The present study considered a single-center cohort of a Multicenter, prospective, randomized controlled study (TrainParc. German Clinical Trials Register, ID: DRKS00010186), which was approved by the local ethic committees of the participating centers (Medical faculty of Cologne, Düsseldorf, Kiel, and Tübingen).

### Cognitive training and physical activity

10 PD-MCI patients underwent cognitive training (CT) and 9 PD-MCI patients underwent physical activity (PA) over a period of six weeks with two sessions per week (90 min each). CT was based on the standardized NEUROvitalis training program [11] that focus on attention, executive functions, memory, and visuocognition. Each session in the program included some of the following components: (1) psychoeducation aimed at creating awareness about memory strategies, healthy cognitive aging and strategies against cognitive decline in PD, (2) group tasks and activity games, (3) individual exercises and (4) homework. The PA intervention aimed to benefit movement abilities of patients. The PA program included: (1) stretching, (2) flexibility, (3) loosening up and (4) relaxation [12].

### Composite score for executive functions and attention

We defined a composite z-score for EF based on normative data corresponding to the following tests: letters/number sequence (WIE), Modified Wisconsin Card Sorting Test (correct categories, perseverative errors, non-perseverative errors) and Regensburg Word Fluency Test (semantic category, phonemic category). For attention, a composite z-score based on the D2 concentration test (error rate and concentration performance) was defined.

### EEG Recording

EEG (128 channels, BrainProducts GmbH (Gilching, Germany) was recorded during resting state (10 min), with eyes open for each participant before and after CT and PA. The recording sampling rate was 5000 Hz and channel impedances were kept in the range 0-20KΩ. FCz served as the reference. No filters were applied during recording. Note that electrophysiological data were collected immediately before and after both training interventions for each participant.

### EEG analysis

#### Preprocessing

EEG was re-referenced to Cz and band pass filtered (0.5–100 Hz). A notch filter (50 Hz) was applied to remove line disturbances. The sampling rate was set up to 512 Hz. Independent component analysis (ICA) was used to remove eye blinks and facial muscle artifacts. The remaining artifacts were visually screened and rejected. For channels with pronounced background noise, we made use of channel interpolation. Artifact-free data were divided into segments of duration 1s. The average length of the EEG recording after data cleaning was 455.4 s (SD 84.2) for pre-intervention and 501.6 s (SD 133.9) for post-intervention.

### Spectral analysis

Power spectrum was calculated by using Fast Fourier Transform with 1 Hz resolution and Hanning window (10% overlap). Focusing on scalp regions on interest, frontal and parietal regions have been considered by previous studies due to their involvement in early and late cognitive dysfunction in PD [13]. Nevertheless, we focus our analysis on the frontal region because of its particular involvement in high cognitive functions specially related to cognitive impairment in PD [14, 15] and PD-MCI [16]. Also, based on brain stimulation studies that stressed the causal role of frontal regions in normalizing the area related to PD-cognitive deficit-related metabolic pattern [17] and cognitive function in PD [18]. Power was extracted from two cortical areas of interest, frontal left (FL) consisting of channels (Fp1, AFp1, AF7, AF3, AFF5h, AFF1h, F1, F3, F5, F7, F9) and frontal right (FR) consisting of channels (Fp2, AFp2, AF8, AF4, AFF6h, AFF2h, F2, F4, F6, F8, F10) by averaging the power of channels corresponding to a specific area and frequency band. The following frequency bands were considered: delta (1-4 Hz), deltatheta (2-7 Hz), theta (4-8 Hz), alpha (8-13 Hz)). All calculations were performed by using Brain Vision Analyzer Version 2.1 (Brain Products GmbH, Gilching, Germany).

### Statistical analysis

#### Joint effect of interventions on cognition

We assessed the joint effect of interventions (pooled data) on EF and attention, e.g. within and between-subjects effect over time, by means of repeated measures ANOVA. All statistical calculations were performed by using SPSS Version 25. The level of significance for all statistical tests was set up to 0.05. Bonferroni correction for multiple comparisons was applied. Effect size for within and between subjects effects were estimated by using the open source software G*power (Version 3.1.9.7) [19].

### Correlation analysis

For the calculation of correlations (Pearson and Spearman, as appropriate regarding distribution of data) between neuropsychological and neurophysiological data, we made use of relative EEG power for areas FL and FR, namely in relation to the ipsilateral and contralateral occipital regions (OL and OR). Note that power ratios were calculated for pre- and post-intervention conditions as well as the difference between pre and post as we targeted power changes over time. Note that pooled data from both intervention were used for the calculation of correlations. We utilized z-standardized values for power analysis.

## Results

**Fig. 1:**
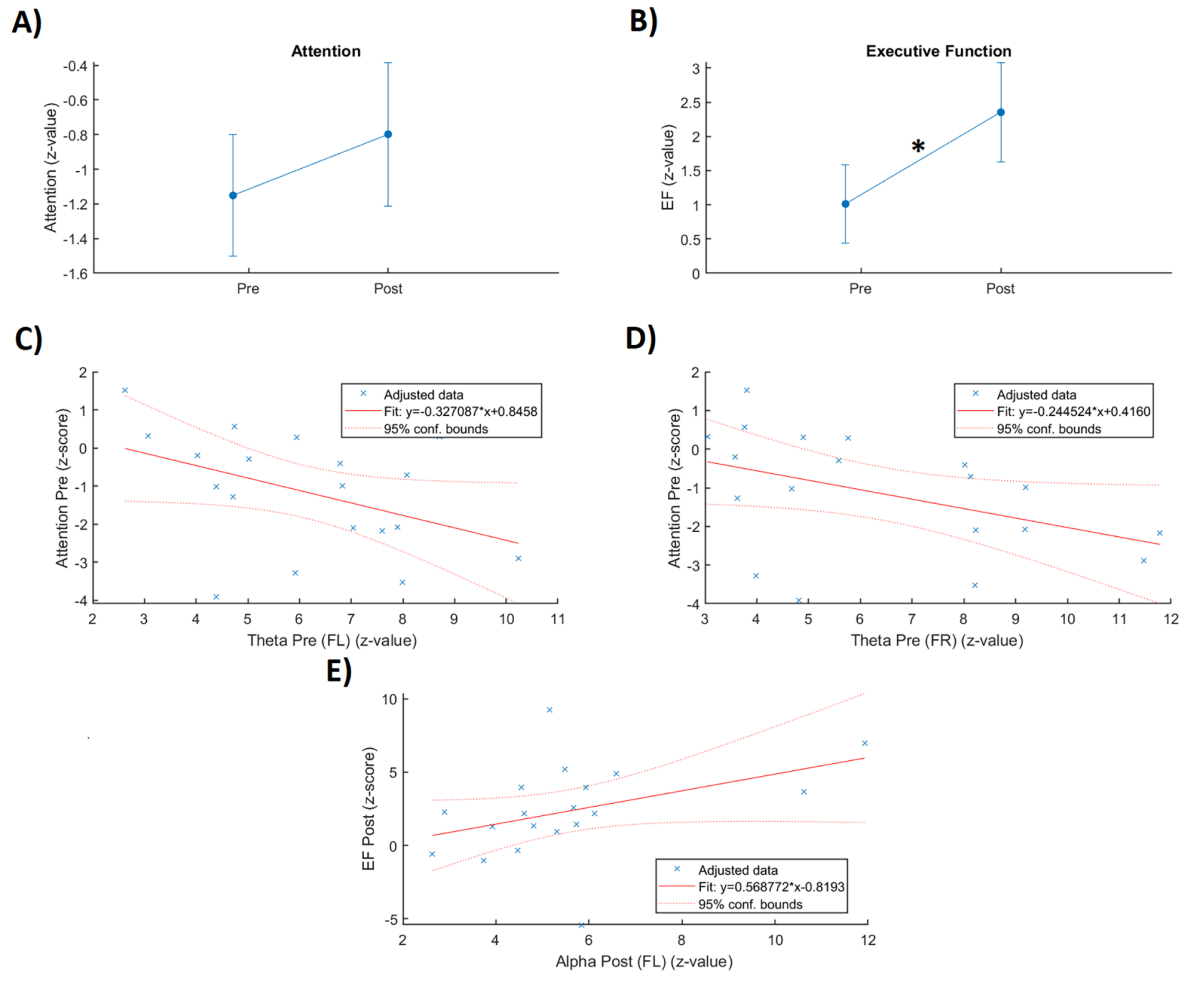
Intervention effects and correlations. **(A)** A trend on the joint effect of interventions on attention was revealed (0.05 < p < 0.1). The graph displays mean and standard error for the composite scores of attention (z-scores); **(B)** Significant joint effect of interventions on executive function (EF) (p < 0.05). The graph displays mean and standard error for the composite scores of EF (z-scores); **(C)** Linear relationship (R^2^ = 0.1895) between attention scores and theta band at FL during pre-intervention; **(D)** Linear relationship (R^2^ = 0.1943) between attention and theta band at FR during pre-intervention; **(E)** Linear relationship (R^2^ = 0.1653) between EF and alpha band at FL during post-intervention

Repeated measures ANOVA revealed a statistical trend towards a positive joint effect of interventions on attention (p = 0.092, 0.05 ≤ p < 0.1) (Fig. 1 (A)). We found a trend toward a within-subjects effect (F(1,18) = 3.171, p = 0.092, partial η^2^_p_ = 0.150, f = 0.420) and a between-subjects effect (F(1,18) = 6.861, p = 0.017, η^2^_p_ = 0.276, f = 0.617) of the factor time.

We found a significant positive joint effect of interventions on EF (p = 0.013) (Fig. 1 (B)). In particular, it was revealed a significant within-subject effect (F(1,18) = 7.589, p = 0.013, η^2^_p_ = 0.297, f = 0.649) and a between-subjects effect (F (1, 18) = 7.592, p = 0.013, η^2^_p_ = 0.297, f = 0.617) of the factor time.

By considering the pooled data, we found a trend in the negative correlation (Pearson) between attention and theta power at FL (r=-0.435, p = 0.063, n = 19) (Fig. 1 (C)) as well as between attention and theta power at FR (r=-0.441, p = 0.059, n = 19) (Fig. 1 (D)) for the condition pre-intervention. A trend in the positive correlation (Pearson) between EF and alpha power at FL (r = 0.407, p = 0.084, n = 19) (Fig. 1 (E)) for the condition post-intervention was indicated. We found a significant negative correlation (Spearman) between attention score and theta power at FR (ρ=-0.497, p = 0.031, n = 19) over time (pre-post).

## Discussion

In agreement with previous reports [12], the joint effect of interventions (CT and PA) was positive on cognitive abilities, e.g. EF and attention, although only the effect on EF was significant. As we utilized data from a single center cohort of the core multicenter study, the within-subject effect on attention was not significant possibly because the cohort was underpowered. In the present analysis, we sought to gain understanding on the neurophysiological manifestation of the joint effect of interventions as reflected in power activity of resting state EEG at frontal brain regions.

Our correlation results point in the direction of a relevant role of resting EEG activity as biomarker of the joint effect of interventions on cognition. Although, the negative relationship (pre-intervention) between attention and theta power at FL and FR as well as the positive relationship (post-intervention) between EF and alpha at FL were not significant, it is observed that 55.56%, 52.63% and 68.42% respectively of patients have a 95% probability that the true linear regression line of the population will lie within confidence interval of the regression line calculated from the sample data. It was also revealed a significant negative relationship (Spearman) between attention and theta over time (post-pre), which indicates that a high attention change would be possibly accompanied with low theta change (post-pre). Interestingly, previous studies reported abnormal functional connectivity patterns in theta band and lower levels of alpha as characteristic in PD-MCI [11, 12]. The fact that PA as part of the join effect of interventions led to improved cognitive abilities, is consistent with previous studies reporting that PA stabilizes disease progression in relevant sensorimotor networks while enhancing cognitive performance [7]. Thus, the present findings support the role of the joint effect of interventions in regulating the power in theta and alpha bands that led to the indicated cognitive improvement (Fig. 1 (A) and (B)).

Based on a recently reported interaction between CT and PA, the present exploratory study used pooled data from both interventions not only to increase statistical power, but also to provide a proof of principle for the utility of resting state EEG as a neurophysiological biomarker of intervention’s effect on cognition in PD-MCI. Note that pooling data was meaningful concerning the statistical effect of joint interventions on EF and the revealed significant relationship between attention and theta band over time. In turn, such observations may be useful concerning the design of future studies and testing of new hypothesis.

Limitations of the present study include a small sample size, which affects the statistical power of our analysis and prevents deeper interpretability on the relationship between theta and alpha band and cognitive improvement induced by both interventions. In a future study, we will incorporate patients from other participant centers, which is also essential for EEG-network analysis.

## Conclusion

Our results support the role of theta and alpha power at frontal areas as a biomarker for the therapeutic joint effect of interventions.

## Data Availability

Data of this study are available from the corresponding author upon reasonable request.

## Abbreviations

PD: Parkinson’s disease
MCI: Mild cognitive impairment
PD-MCI: Parkinson’s disease with mild cognitive impairment
CT: Cognitive training
PA: Physical Activity
MEG: Magnetoencephalogram
EEG: Electroencephalogram
UPDRS: Unified Parkinson’s disease rating scale
MoCA: Montreal Cognitive Assessment
SCI: Subjective cognitive impairment questionnaire
BDI II: Beck Depression Inventory II
GSE: General Self-Efficacy Questionnaire
